# Slow-Relaxation Behavior of a Mononuclear Co(II) Complex Featuring Long Axial Co-O Bond

**DOI:** 10.3390/nano12040707

**Published:** 2022-02-21

**Authors:** Zhengyao Xia, Yan Li, Cheng Ji, Yucheng Jiang, Chunlan Ma, Ju Gao, Jinlei Zhang

**Affiliations:** 1 Jiangsu Key Laboratory of Micro and Nano Heat Fluid Flow Technology and Energy Application, School of Physical Science and Technology, Suzhou University of Science and Technology, Suzhou 215009, China; zhengyaoxia@163.com (Z.X.); jcspst@126.com (C.J.); wlxmcl@mail.usts.edu.cn (C.M.); 2 School of Environmental Science and Engineering, Suzhou University of Science and Technology, Suzhou 215009, China; ly15822867871@163.com; 3 School of Optoelect Engn, Zaozhuang University, Zaozhuang 277160, China

**Keywords:** single-ion magnet, high effective energy barrier, long axial bond

## Abstract

Co(II) mononuclear complex with different coordination geometry would display various of field-induced single-ion magnet (SIM) behaviors. Here, we identify a field-induced single-ion magnet in a mononuclear complex Co(H_2_DPA)_2_·H_2_O (H_2_DPA = 2,6-pyridine-dicarboxylic acid) by the hydrothermal method. The long axial Co-O coordination bond (Co1‧‧‧O3) can be formed by Co1 and O3. Therefore, Co(II) ion is six-coordinated in a distorted elongated octahedron. AC magnetization susceptibilities show that the effective energy barrier is up to 43.28 K. This is much larger than most mononuclear Co(II). The distorted elongated octahedron caused by the axial Co-O coordination bond is responsible for the high effective energy barrier. The distribution of electron density in Co1 and O3 atoms in the long axial bond would influence the magnetic relaxation process in turn. Our work deepens the relationship between the effective energy barrier and the weak change of ligand field by long axial bonds, which would facilitate constructing SIM with high energy temperature.

## 1. Introduction

The first single-ion magnet (SIM) [Tb(III)Pc_2_] was reported in 2003 [1]. From then, a lot of lanthanide-based SIMs were prepared and studied owing to their large single-ion magnetic anisotropies which may lead to higher energy barriers [2,3,4,5,6,7]. Owing to the valence orbitals of transition metal ions, the 3d orbital angular momentum could be quenched easier than that of lanthanide by the ligand field. Therefore, the magnetic anisotropy of a transition metal based complex could be greatly owned to the second-order spin–orbit coupling in the mixed states between excited and ground states [8,9]. Among the transition metal ions, Co(II) ion has been mostly researched owing to its strong magnetic anisotropy. In addition, Co(II) ion based complex show numerous coordination geometries such as two coordination [10], three coordination [11], four coordination [12,13], five coordination [14], six coordination [15,16,17], seven coordination [18] and eight coordination [19].

Until now, most reported field-induced SIM behavior is shown in the six-coordinated Co(II) mononuclear complex. As the central symmetry of paramagnetic complex plays an important role in the slow relaxation. Their magnetostructural relationships have been studied deeply [8]. Song’s group has even reported the relationship between the coordination geometry of Co(II) ions and slow relaxation in the six-coordinated Co(II) complex in 2018 [20]. However, there are few studies on SIMs with a distorted elongated octahedron coordination environment of Co(II) ions in the complex. The effects of the weak change of the ligand field on the slow relaxation and the positive/negative value of *D* are still insufficient in Co(II) complexes [7,21,22,23,24,25,26,27,28,29,30,31,32,33,34,35,36,37]. Therefore, it is necessary to study the relationship between magnetic characteristics and the long axial Co-O bond in Co(II) complexes with SIMs behavior. Here, we synthesized a six-coordination mononuclear cobalt complex Co(H_2_DPA)_2_(H_2_O) (1, H_2_DPA = 2,6-pyridine-dicarboxylic acid) with a distorted elongated octahedron environment, which shows field-induced slow-relaxations. We demonstrate that the distorted octahedron caused by the long axial Co-O bond (Co1‧‧‧O3) could play an important role in increasing the effective energy barrier of SIM, which is about 43.28 K.

## 2. Materials and Methods

First, 2.0 mmol piperazine (172.28 mg) in 10 mL C_2_H_5_OH was added dropwise to 0.5 mmol CoCl_2_·6H_2_O (118.97 mg) and 0.5 mmol H_2_DPA (83.56 mg) in 20 mL H_2_O. Then, by filtering and evaporating naturally at room temperature for three days [38,39,40], we obtained crystals with the distorted octahedral geometry named complex **1**. The yield of the complex crystal is about 40%.

Crystallographic data for the selected complex **1** crystal were obtained at 296 K through Bruker D8 Venture [41,42,43]. The structure data were uploaded in a CIF file. Detailed structural analysis is displayed in Table S1 in the Supplementary Materials. The related distances and angles are listed in Table S2. The experimental XRD pattern was collected by Bruker D8 Advanced, and the simulated XRD pattern was fitted by the software of Diamond. The magnetization was measured from 1.8 to 300 K by SQUID (MPMS-XL 7). Then, measurements of isothermal magnetization were carried out from 0 to 7 T. The corresponding AC susceptibility of the same crystals was also measured at different external fields, with different frequencies from 1 to 999 Hz [44,45]. 

## 3. Results and Discussion

### 3.1. Structure

**Figure 1:**
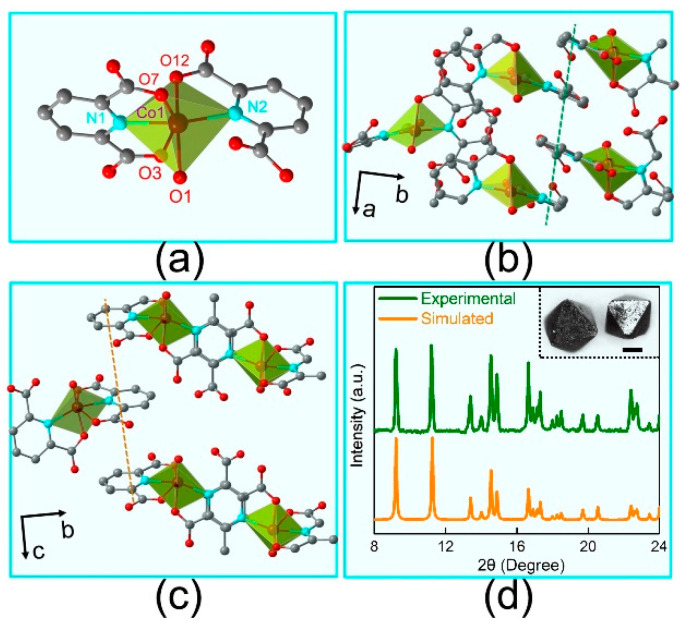
Atomic structure of **1**. (**a**) The distorted elongated octahedron centered with a Co(II) ion; (**b**,**c**) two different π‧‧‧π stackings along ***a*** axis (**b**) and ***c*** axis (**c**) in **1** respectively; (**d**) XRD spectra of experimental and simulated **1**. The inset is the morphology of crystal complex **1**. The scale bar is 0.2 mm.

By the analysis of single crystal X-ray diffraction, this Co(II) based complex **1** is monoclinic with a space group of P2_1_/n. Every Co(II) ion is six-coordinated in a distorted elongated octahedron (Table S3) with two H_2_DPA molecules and one water (Figure 1a). All the square positions of the distorted elongated octahedron are occupied by O7, O12, N1 and N2 respectively, where two oxygen and two nitrogen atoms are from H_2_DPA ligands. The last oxygen atom named O1 is from water. Obviously, all the Co-O/N coordinated bonds are in the range for a high spin of Co(II) ion. Among them, the Co-O bond lengths are 2.0539, 2.0620, and 2.1042 Å, respectively, and Co-N ones are 2.0719 and 2.1423 Å for **1**, respectively. Note that the adjacent Co1‧‧‧O3 distance is 2.4265 Å named as the long axial bond, which is much longer than that in the similar mononuclear Co complex. There is a strong π‧‧‧π stacking (3.4199 Å) between the nearest molecules along the ***a*** axis as shown in Figure 1b. Then the nearest two molecules form a one-dimensional (1D) chain along ***c*** axis by π‧‧‧π stacking (3.5731 Å), therefore constructing its three-dimensional structure of complex **1** (Figure 1c). The morphology of complex **1** crystals showing distorted octahedral geometry is displayed in the inset of Figure 1d. The crystal edge length of the geometry is about 0.5 mm. Then XRD spectra can be acquired from the grinded powders of the selected complex crystals in Figure 1d (olive line). By contrasting the simulated spectra (orange line), we can confirm the structure analysis of complex **1**.

### 3.2. Magnetic Properties

**Figure 2:**
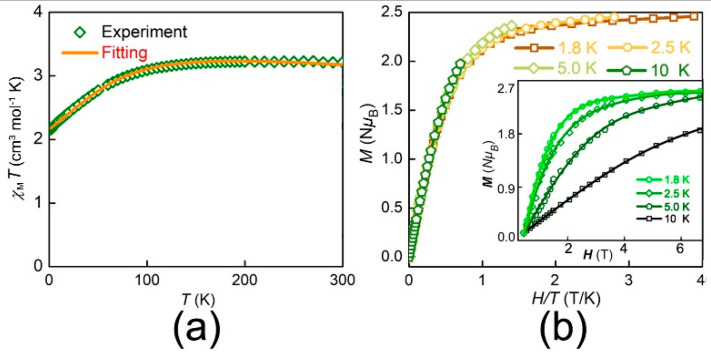
(**a**) Temperature-dependent *χ*_M_*T* for **1.** The solid line is fitted by PHI; (**b**) Isothermal reduced magnetization at different temperatures for **1**. Insert: Experimental M(H) plots at different temperatures for **1**. Solid lines represent the best fit.

Static direct-current (DC) magnetization measurements were performed ranging from 1.8 to 300 K under an external magnetic field of 1 kOe on the polycrystalline samples of **1**. At 300 K, the complex **1** shows its *χ*_M_*T* value of about 3.21 cm^3^ K mol^−1^ in Figure 2a. This *χ*_M_*T* value is much larger than that of isolated high spin Co(II) (S = 3/2 and g = 2.0) of 1.87 cm^3^ mol^−1^ K. The large *χ*_M_*T* in complex usually originates from spin-orbit coupling [8]. The *χ*_M_*T* values first decrease gradually as the room temperature decreases to 150 K, then shows a sharp decrease as the temperature lowers, finally reaching the ultimate values of 2.16 cm^3^ mol^−1^ K at 1.8 K. This is commonly owned to the depopulation of Kramers’ excited state levels (*M*_J_ = ±3/2 and ±5/2). Therefore, M(H) characteristics of complex **1** at 1.8, 2.5, 5.0 and 10 K were measured under a field ranging from 0 to 7 T in the inset of Figure 2b. At 1.8 K, complex **1** increases continuously to 2.46 N*μ*_B_ at 7 T. This non-saturation with a high-field further shows an obviously magnetic anisotropy of complex **1**. To further confirm the magnetic anisotropy of complex **1**, the corresponding reduced magnetization was shown in Figure 2b. The magnetization of 2.46 N*μ*_B_ at 7 T is as expected for an anisotropic ion. By utilizing the PHI program, the anisotropy parameters of complex **1** can be quantified from the temperature and field-dependent magnetization data [46]. Good fits could be obtained by using the following spin Hamiltonian:

$$\hat{H}=D\left[{\hat{S}}_{z}^{2}-\frac{S\left(S+1\right)}{3}\right]+E\left({\hat{S}}_{x}^{2}-{\hat{S}}_{y}^{2}\right)+g\mu_{B}SH,$$

where *D* is the axial parameter and *E* is parameter of rhombic zero-field-splitting parameter respectively, *S* is the spin projection and the last term is the Zeeman. The best fits of the reduced magnetization data show *D* = 67.63(22) cm^−1^, *E* = −16.29(15) cm^−1^, *g* = 2.77(3) and *TIP* = 1.21 × 10^−4^ for complex **1**. This positive *D* value can be mainly owned to the coupled states between ground and excited states.

**Figure 3:**
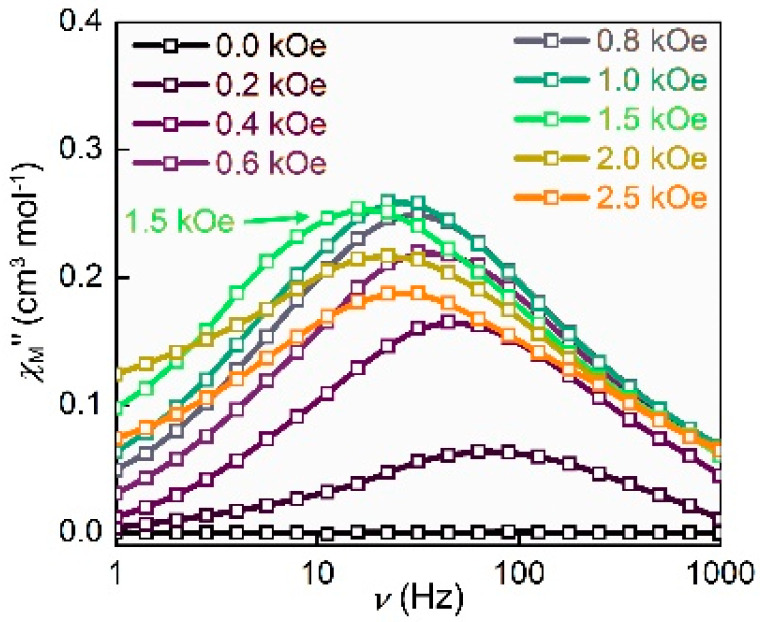
Isothermal field-dependent magnetic characteristics performed on polycrystalline sample of complex **1** at 2 K.

**Figure 4:**
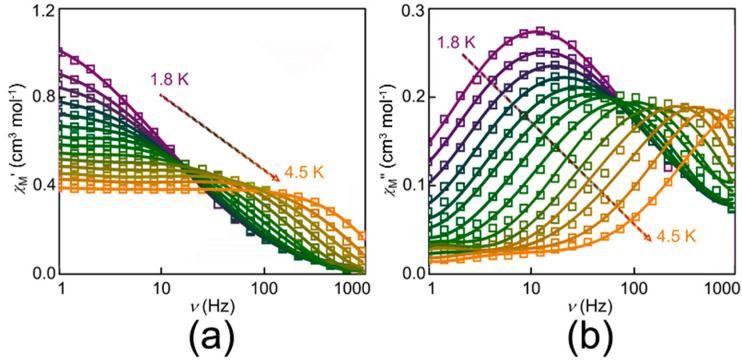
Frequency-dependent *χ*_M_*′* (**a**) and *χ_M_″* (**b**) AC susceptibilities in *H*_DC_ = 1.5 kOe for complex **1**.

**Figure 5:**
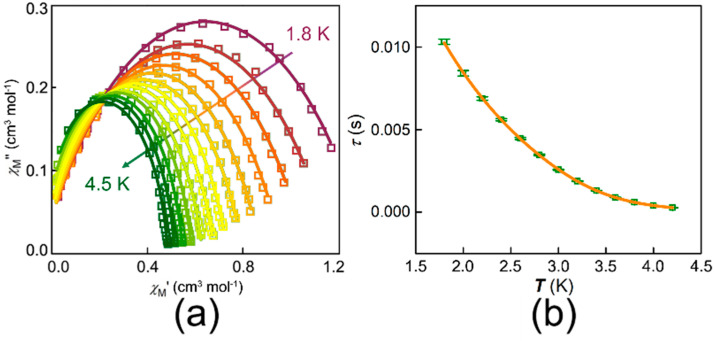
(**a**) The Cole-Cole curves of complex **1**. Solid lines are fitted by CCFIT2; (**b**) Plot of *τ*_0_ versus *T* for complex **1**, where the orange solid line represents the fitted results using CCFIT2.

To probe the magnetic relaxation dynamics, the magnetic susceptibilities were measured. For complex **1**, there is no obvious signal of *χ*_M_*″* of AC susceptibility (out-of-plane) at 2 K. However, typical signals of *χ*_M_*″* can be observed directly, when an external DC magnetic field is applied. Figure 3 shows the typically frequency-dependent AC magnetic susceptibility under different magnetic fields from 0 to 2.5 kOe at 2.0 K. Such behavior is associated with the appearance of quantum tunneling of the magnetization (QTM) [12]. This result indicates that each molecule of complex **1** containing a cobalt behaves as a field-induced SIM. To further explore the magnetostructural correlation, 1.5 kOe was the best to be chosen to measure the dynamic magnetization due to the longest relaxation time. The variable frequency *χ*_M_*′* and *χ*_M_*″* for **1** at temperatures ranging from 1.8 to 4.5 K are shown in Figure 4a,b, respectively. Obvious frequency-dependent out-of-phase peaks could be observed at the temperature from 1.8 to 4.5 K in Figure 4b. Such behavior is commonly related to the super-paramagnet-like slow magnetic relaxation in a typical SIM. Then, plots of *χ*_M_*″* vs. *χ*_M_*′* (Cole–Cole plot) for complex **1** were fitted well by using the CCFIT2 program and the modified Debye function (Figure 5a) [47]. From the Arrhenius-like diagrams in Figure 5b and Table S4, relaxation times (*τ*_0_) and effective barrier energies could be calculated. Equation (1) was applied to fit, which contains three relaxation processes, where *A* means the direct process, *B* indicates the process of Raman, *U*_eff_ means the effective energy barrier of magnetization reversal. According to these corresponding data, these fitted parameters are *A* = 43.54 K^−1^ S^−1^, *B* = 1.22 × K^−4.67^ S^−1^, *n* = 4.67 respectively. We can also gain that *τ*_0_ is 2.11 × 10^−8^ s of the slow relaxation time. *U*_eff_ shows a large value of about 43.28 K. *U*_eff_ is much larger than the ones in most mononuclear Co complexes ever reported.

*τ*^−1^ = *AT* + *BT^n^* + *τ*_o_^−1^exp(−*U*_eff_/*k*_B_*T*). (1)

Compared with other mononuclear Co(II) complexes [22,27,28,29,30,31,32,33,34,35,36,37], the *χ*_M_*T* value of 3.21 cm^3^ K mol^−1^ in **1** is much larger than other Co based complexes. This result may be due to the long axial Co-O bond formed by Co1 and O3 (Co1‧‧‧O3 distance is 2.4265 Å), which certainly is not a coordination bond. Owing to the long axial Co-O bond, the Co(II) complex shows the distorted elongated octahedron. Therefore, the larger *χ*_M_*T* values look like the one in six coordination Co(II) complexes. On the other hand, the distorted elongated octahedron could further change the electron density between Co(II) and O ions. We applied the first-principles calculations based on the density functional theory (DFT) to evaluate the distribution of spin state with different length of axial Co-O bond in Figure S1. There is a strong relationship between the distribution of spin state and the length of axial Co-O bond. The complex with longer axial Co-O bond, shows the existence of spin states. Furthermore, the charge density difference was calculated to distinctly analyze the charge variation with different spin states. Figure S2 and Figure S3 shows that Co1 donates more charge and O3 accepts more charge with a spin up state (longer axial Co-O bond) than those without a spin state (shorter axial Co-O bond). Meanwhile, the charge transfer is relatively less between spin up and spin down states. Therefore, we conclude that the long axial Co-O bond in complex **1** may play an important role in increasing the effective energy barriers up to 43.28 K. However, the effective energy barriers of most mononuclear Co(II) complexes are lower than 20 K.

## 4. Discussion

We report a mononuclear Co(II) complex with the distorted elongated octahedral geometry. The result value of *D* obtained by fitting the DC susceptibility is positive, indicating that complex **1** is an easy-plane complex. Furthermore, complex **1** is a typical SIM representing field induced slow-relaxation. AC magnetic susceptibility measurement results confirm that complex 1 is a field-induced SIM with a high effective energy barrier *U_eff_* of above 43.28 K and relaxation time *τ*_0_= 2.11 × 10^−8^ s. By DFT calculation, this high effective energy barrier and high *χ*_M_*T* value of complex 1 could be originated from the distorted elongated octahedron environment caused by the long axial Co-O bond. These results may offer a different strategy to improve blocking the temperature of a single-ion magnet.

## Data Availability

Not applicable.

## Supplementary Materials

The following supporting information can be downloaded at: https://www.mdpi.com/article/10.3390/nano12040707/s1, Table S1: Crystallographic data and structural refinement parameters for complex**1**; Table S2: Bond lengths [Å] and angles [deg] for **1**; Table S3: Deviation parameters calculated by SHAPE from each ideal polyhedron for complex Co1; Table S4: The fit parameters of the analyses of the ac susceptibilities of 1 under 1.5 kOe bias DC field; Figure S1: Structures for complex **1** with different length of axial Co-O bonds. Figure S2: Charge density difference between different spin states. Figure S3: The standard distribution of electron density determined by single-crystal X-ray diffraction; Structure S1: crystallographic CIF. References [48,49,50,51] are cited in the Supplementary Materials.
