# Supplementary material for: Slow-Relaxation Behavior of a Mononuclear Co(II) Complex Featuring Long Axial Co-O Bond
Source: Nanomaterials (Basel). 2022 Feb 21;12(4):707. doi: 10.3390/nano12040707 (PMC8875892; doi:10.3390/nano12040707)
Supplement: Supplementary file 1 [file nanomaterials-12-00707-s001.zip › Supplementary Materials-Nanomaterials.pdf]

Slow-Relaxation Behavior of a Mononuclear Co(II) Complex Featuring Long Axial Co-O Bond

Zhengyao Xia [1], Yan Li [2], Cheng Ji [1], Yucheng Jiang [1,*], Chunlan Ma [1], Ju Gao [1,3,*] and Jinlei Zhang [1,*]

[1] Jiangsu Key Laboratory of Micro and Nano Heat Fluid Flow Technology and Energy Application, School of Physical Science and Technology, Suzhou University of Science and Technology, Suzhou 215009, China; zhengyaoxia@163.com (Z.X.); jcspst@126.com (C.J.); wlxmcl@mail.usts.edu.cn (C.M.);
[2] School of Environmental Science and Engineering, Suzhou University of Science and Technology, Suzhou 215009, China; ly15822867871@163.com
[3] Sch Optoelect Engn, Zaozhuang Univ, Zaozhuang 277160, China
* Correspondence: jyc@usts.edu.cn (Y.J.); jugao@hku.hk (J.G.); zhangjinlei@usts.edu.cn (J.Z.)

Supplementary Text

Methods

Computational details

The first-principles calculations were performed with the Perdew-Burke-Ernzerhof (PBE) functional [48] in the Vienna Ab initio Simulation Package (VASP) [49,50]. The Hubbard U correction (5.6 eV) [51] for Co was adopted to accurately describe the strong correlation of d-electrons. The potentials of Co, O, H, N and C were used the elements evaluates. The plane-wave cutoff energy and the Monkhorst-Pack grid were set as 500 eV and 3 × 1 × 2, respectively. For the structural relaxations, the energy and stress convergences were 1×10^{-4} eV and 0.01 eV $Å^{-1}$, respectively. All the spin up, spin down and no spin were taken into consideration to describe the distribution of spin density in the molecule. The charge density differences were calculated to distinctly analyze the charge variation with different spin states.

DFT calculations

The first-principles calculations based on the density functional theory (DFT) were carried out to evaluate the distribution of spin density in the molecule. DFT calculations show that the long

axial Co-O bonds were 2.46461, 2.31959 and 2.46671 Å for spin up, spin down and no spin three states, respectively (Figure S1). These results indicate that there is a strong relationship between the distribution of spin density and the bond length and strength. The spin states could existence in complex with longer axial Co-O bond. Furthermore, the charge density difference was calculated to distinctly analyze the charge variation with different spin states. The charge density difference calculations exhibit that Co1 donates more charge and O3 accepts more charge with spin up state than these without spin state (Figure S2a). However, the charge transfer is relative less between spin up and spin down states (Figure S2b). These calculations visualize the distribution of electron density in a molecule and explain the relationship between the spin states and the long axial Co-O bond.

Table S1 Crystallographic data and structural refinement parameters for complex **1**

	**1**
Formula	$C_{14}H_{16}CoN_2O_{12}$
f w	463.22
T / K	296(2)
λ / Å	0.71073
Crystal system	Monoclinic
Space group	$P2_1/n$
a / Å	6.9863(3)
b / Å	23.7443(11)
c / Å	10.6564(5)
α / º	90
β / º	100.4560(10)
γ / º	90
V / Å^3	1738.38(14)
Z	4
D_c / g cm^{-3}	1.770
μ / mm^{-1}	1.061
F(000)	948
θ / °	2.592 to 27.526
Reflns collected	15385
Reflns unique	3989
R_{int}	0.0284
GOF on F^2	1.085
R_1 [I> 2σ(I)][a]	0.0278
wR_2 [I>2σ(I))][b]	0.0646
R_1 (all data)[a]	0.0334
wR_2 (all data)[b]	0.0667
Largest diff. Peak, hole / (e Å^{-3})	0.353 and -0.441

[a]$R_1 = \Sigma||Fo| - |Fc||/\Sigma|Fo|$; [b]$wR_2 = [\Sigma w(Fo^2 - Fc^2)^2/\Sigma w(Fo^2)^2]^{1/2}$.

Table S2 Bond lengths [A] and angles [deg] for **1**

Co1-O12	2.0539(12)	Co1-O1	2.1042(12)
Co1-O7	2.0620(11)	Co1-N2	2.1423(13)
Co1-N1	2.0719(13)		
O12-Co1-O7	160.52(5)	N1-Co1-O1	77.05(5)
O12-Co1-N1	94.02(5)	O12-Co1-N2	100.23(5)
O7-Co1-N1	85.50(5)	O7-Co1-N2	77.37(5)
O12-Co1-O1	103.16(5)	N1-Co1-N2	161.97(5)
O7-Co1-O1	95.73(5)	O1-Co1-N2	109.94(5)

Table S3 Deviation parameters calculated by SHAPE from each ideal polyhedron for complex **Co1**. The best matches are displayed in red showing the optimal geometry is distorted elongated octahedron. HP-6 = Hexagon, PPY-6 = Pentagonal pyramid, OC-6 = Octahedron, TPR-6 = Trigonal prism, JPPY-6 = Johnson pentagonal pyramid (J2)

Geometry	Symmetry	Co1
HP-6	D6h	32.486
PPY-6	C5v	23.809
**OC-6**	**Oh**	**2.918**
TPR-6	D3h	11.638
JPPY-6	C5v	27.641

Table S4 The fit parameters obtained from analyses of the AC susceptibilities of **1** under 1.5 kOe bias DC field.

T / K	χ_T / cm^3 mol^{-1}	χ_S / cm^3 mol^{-1}	τ / s	τ_{erro}	a
1.8	1.19515	0.08933	0.01031	1.61E-04	0.38241
2.0	1.07401	0.08874	0.0084	1.69E-04	0.37701
2.2	0.98193	0.09282	0.00688	1.06E-04	0.34843
2.4	0.90277	0.09348	0.0056	6.45E-05	0.33005
2.6	0.82899	0.09539	0.00446	5.99E-05	0.29925
2.8	0.76781	0.09508	0.00346	4.42E-05	0.27249
3.0	0.71516	0.09375	0.00257	2.86E-05	0.23899
3.2	0.67146	0.09108	0.00188	2.34E-05	0.20908
3.4	0.63058	0.08489	0.0013	1.59E-05	0.18941
3.6	0.59473	0.08326	8.87E-04	1.17E-05	0.155
3.8	0.0564	0.07596	5.97E-04	7.39E-06	0.13969
4.0	0.0537	0.07385	4.05E-04	5.50E-06	0.11764
4.2	0.51216	0.06573	2.70E-04	6.66E-06	0.10855

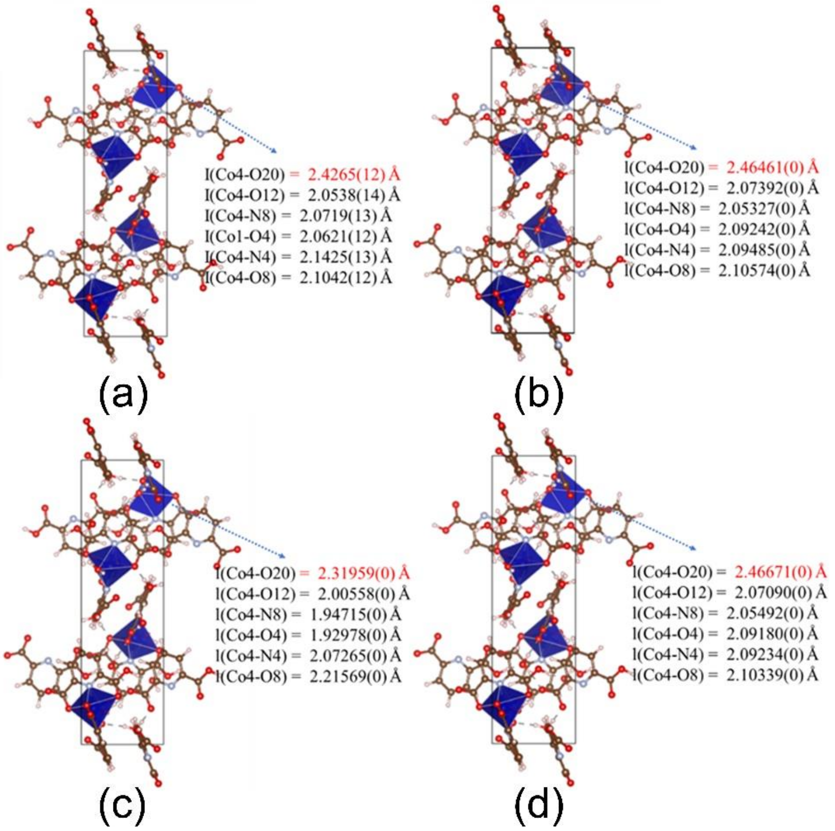

Figure S1 Structures for Co(H_2DPA)$_2$(H_2O) with different length of axial Co-O bonds. (**a**) The experimental structure by single-crystal X-ray diffraction analysis. (**a**), (**b**) and (**c**) are the density functional theory (DFT) calculations: spin up (**b**), no spin (**c**) and spin down (**d**).

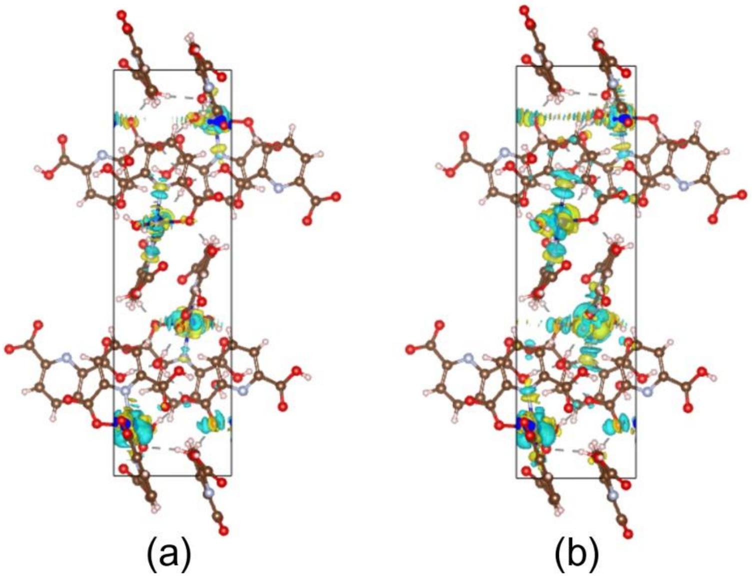

Figure S2 Charge density difference between different spin states. The distribution of electron density difference between (**a**) spin up and no spin states, (**b**) spin up and spin down states. The yellow and cyan areas represent the increased charge and the decreased charge, respectively.

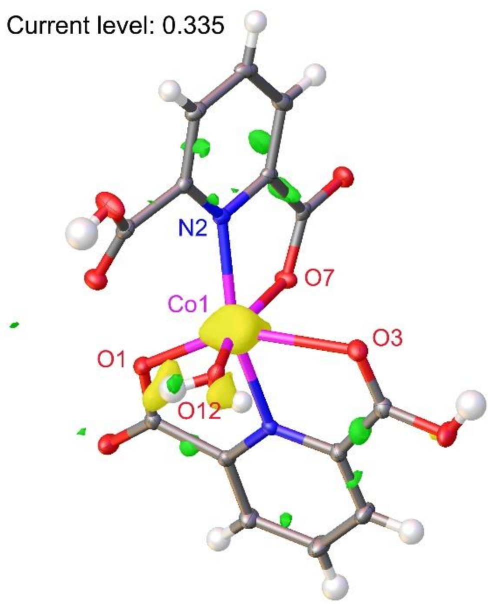

Figure S3 The standard distribution of electron density determined by single-crystal X-ray diffraction.

References

48. Perdew, J. P.; Burke, K.; Ernzerhof, M. Generalized gradient approximation made simple. *Phys. Rev. Lett.* **1996**, 77, 3865-3868.

49. Hohenberg, P.; Kohn, W. Inhomogeneous electron gas. *Phys. Rev. B* **1964**, *136*, B864-B865.

50. Kresse, G.; Furthmuller, J. Efficiency of ab-initio total energy calculations for metals and semiconductors using a plane-wave basis set. *Comput. Mater. Sci.* **1996**, *6*, 15-50.

51. Zhou, F.; Cococcioni, M.; Marianetti, C. A.; Morgan, D.; Ceder, G. First-principles prediction of redox potentials in transition-metal compounds with LDA + U. *Phys. Rev. B: Condens. Matter Mater. Phys.* **2004**, *70*, 235121.
